# Environmental determinants of cardiovascular-kidney-metabolic health: interactive roles of air pollution, heat waves, and green spaces

**DOI:** 10.7189/jogh.16.04073

**Published:** 2026-02-27

**Authors:** Ao Li, Chuangsen Fang, Yanming Li

**Affiliations:** Department of Respiratory and Critical Care Medicine, Beijing Hospital, National Centre of Gerontology, Institute of Geriatric Medicine, Chinese Academy of Medical Sciences, Beijing, China

## Abstract

**Background:**

Cardiovascular-kidney-metabolic (CKM) syndrome, characterised by linked metabolic, renal, and cardiovascular dysfunctions, confers high cardiovascular risk and represents a major public health concern in China. Environmental exposures such as air pollution, heat waves, and limited greenness may exacerbate cardiometabolic burden. Limited studies evaluated their independent and interactive effects on CVD risk among individuals with CKM syndrome.

**Methods:**

We assessed city-level annual environmental exposures using data from the China Health and Retirement Longitudinal Study 2011–2018. Specifically, we evaluated the effects of PM_1_, PM_2.5_, PM_10_, nitrogen dioxide (NO_2_), and heat waves on CVD incidence using Cox proportional hazards models, and examined multiplicative interactions via product terms. We quantified additive interactions using the relative excess risk due to interaction.

**Results:**

Exposure to air pollution (PM and NO_2_) and heat waves was associated with higher CVD risk, whereas a higher normalised difference vegetation index (NDVI) was protective from CVD. We observed significant interactions on both multiplicative and additive scales. Specifically, the joint association between PM and heat waves was greater than the sum of their individual risks (a positive additive interaction), suggesting that heat waves may exacerbate the risk associated with air pollution. Conversely, negative interaction terms involving NDVI suggested that higher greenness might modify the association between PM and CVD risk. These patterns were more pronounced in participants with advanced CKM stages.

**Conclusions:**

Our findings provide novel evidence that multiple environmental stressors interact to influence CVD risk in the CKM population, highlighting the importance of integrated environmental and public health strategies.

Cardiovascular-kidney-metabolic (CKM) syndrome represents the clinical manifestation of interconnected metabolic, renal, and cardiovascular dysfunctions, posing a major public health concern globally and in China. While recent studies have explored lifestyle and pharmacological modifiers, the environmental dimension of CKM progression remains underexplored, despite its recognition by the American Heart Association [1,2].

Rapid urbanisation in China has intensified environmental challenges of air pollution, reduced greenness, and heat waves, each of which contributes to cardiovascular and metabolic burdens. Air pollution ranks among the leading global causes of morbidity and mortality [3], while excessive heat exposure further worsens cardiovascular risks, particularly in vulnerable populations with cardiometabolic conditions [4–9]. Residential greenness, typically measured by the normalised difference vegetation index (NDVI), has been linked to lower all-cause and cardiopulmonary mortality and may buffer the adverse effects of air pollution and heat. However, its role in the CKM population and its interactions with other exposures remain unclear.

There is a paucity of evidence on how environmental factors influence individuals with CKM [10]. Previous epidemiological studies have primarily examined single exposures and focused on specific disease domains, such as cardiovascular disease (CVD), diabetes, or chronic kidney disease. Indeed, humans are simultaneously exposed to a wide range of environmental factors that often interact, either synergistically or antagonistically. To date, little is known about the interactions among multiple environmental factors in this specific CKM population. Clarifying both the independent and interactive effects of these exposures has become increasingly urgent amid rapid urbanisation and the growing disease burden in China. This would not only capture the complexity of environmental influences by identifying interactions among exposures, but also provide a stronger basis for designing targeted interventions and regulations. Therefore, using data from the China Health and Retirement Longitudinal Study (CHARLS), we investigated the interactive effects of air pollution, heat waves, and green space on the incidence of CVD among adults at CKM stages 0–3, to generate evidence on environmental determinants that would inform CKM management.

## METHODS

This study involves secondary analysis of a large, publicly available data repository. We report this work in accordance with the Journal of Global Health’s Guidelines for Reporting Analyses of Big Data Repositories Open to the Public (GRABDROP) [11] (Table S1 in the **Online Supplementary Document**).

### Study population

We obtained participants’ data from the CHARLS, a nationally representative survey that uses multistage, stratified sampling to collect population-level health data across 28 provinces in China. We analysed data from 2011 to 2018 on a cohort of participants aged ≥45 years who had sufficient information to determine CKM stage (0–3), were free of CVD at baseline, and had geocoded residential addresses for environmental exposure assessment. A total of 5289 participants from CHARLS met these criteria and provided written informed consent (Figure S1 in the **Online Supplementary Document**). The ethics committee of Peking University approved the CHARLS (IRB00001052-11015).

### Assessment of exposure variables

We obtained ambient air pollution levels from the China High Air Pollution data set. Detailed methods for air pollution estimation have been described elsewhere [12–15]. Briefly, we used an integrated approach that combined satellite-based spatiotemporal models, ground-level monitoring data, meteorological parameters, and land-use regression to assess annual air pollutant concentrations at high spatial resolution. Furthermore, we predicted daily concentrations of PM_1_ and PM_2.5_ using satellite aerosol optical depth retrievals, adjusted for local meteorological conditions (*e.g.* wind, temperature, and humidity) and land-use characteristics (*e.g.* road networks and industrial activity). We retrieved concentrations of PM_10_, nitrogen dioxide (NO_2_), and ozone (O_3_) directly from China’s national air quality monitoring network.

We retrieved city-level temperature data from the National Centres for Environmental Information. Further, we defined heat waves as periods of ≥3 consecutive days during which the daily mean temperature exceeded the 92.5th percentile of the annual mean temperature. Additionally, we tested alternative percentile thresholds in sensitivity analyses.

We assessed residential greenness using the NDVI, with higher values indicating denser vegetation. We obtained the NDVI data from the Resource and Environmental Science and Data Centre and used values >0 to represent vegetated areas. Furthermore, we calculated long-term exposure levels as the mean of annual estimates during follow-up and linked them to each participant’s residential address and informed consent.

### Definitions of CKM syndrome stages 0–3

We classified CKM stages using objective biomarkers and self-reported data from the CHARLS, in accordance with the American Heart Association guidelines. The stages were defined as follows: stage 0 (no risk factors; absence of any CKM risk factors), stage 1 (excess adiposity; abdominal obesity with waist circumference thresholds of 90 cm for men and 80 cm for women), stage 2 (metabolic risk or CKD; presence of one or more metabolic abnormalities or moderate-to-high risk chronic kidney disease, such as elevated triglycerides, reduced high-density lipoprotein cholesterol, diabetes, hypertension, or impaired kidney function), and stage 3 (subclinical CVD; representing high predicted risk or subclinical disease using the American Heart Association Predicting Risk of Cardiovascular Disease Events equations (Table S2 in the **Online Supplementary Document**).

### Definition of CVD

We defined CVD based on self-reported medical diagnoses in response to the following questionnaire items: ‘Have you ever been diagnosed with a heart attack, coronary heart disease, angina, congestive heart failure, or other heart problems by a doctor since the last interview?’ and ‘Have you ever been diagnosed with a stroke by a doctor since the last interview?’ We classified participants as having CVD if they answered positively to at least one of these questions (Table S3 in the **Online Supplementary Document**).

### Covariates

We selected the following covariates based on prior literature: demographics (age, sex, residence, education, and region), lifestyle factors (smoking, drinking, and cooking fuel use), and clinical factors (hypertension, diabetes, hyperlipidaemia, waist circumference, body-mass index (BMI), triglycerides, low-density lipoprotein cholesterol (LDL-C), fasting glucose, and systolic and diastolic blood pressure). We adjusted for socioeconomic status by including it as a covariate in the multivariable Cox proportional hazards models. Further, we categorised geographic regions in two ways to address different research questions. First, we classified participants into Eastern, Central, and Western China according to the National Bureau of Statistics economic zones to account for socioeconomic development levels. Second, for subgroup analyses examining climatic and policy differences, we stratified participants into Northern and Southern China based on the geoclimatic boundary defined by the Qinling Mountain and the Huai River Line.

### Data analysis

Among 5289 participants with CKM stages 0–3 at baseline, we classified individuals according to whether they developed CVD during follow-up. We compared baseline characteristics between groups using parametric or non-parametric tests for continuous variables and χ^2^ tests for categorical variables. We assessed relationships among environmental exposures using Pearson correlation analysis.

We implemented two analytic stages. First, using time-varying Cox proportional hazards models, we estimated hazard ratios (HRs) for CVD associated with individual exposures (10 μg/m^3^ increase in air pollutant concentration, a one-day increase in heat-wave duration, and a 0.1-unit increase in NDVI). We applied two models – model 1 (unadjusted) and model 2 (fully adjusted for demographic, behavioural, and socioeconomic covariates). Second, we incorporated cross-product terms into the models to evaluate potential interactive effects of air pollution, heat waves, and green space exposure on CVD incidence. Specifically, we examined interactions among increases in pollutant concentration and heat wave days, pollutant concentration and NDVI, and heat wave days and NDVI. It should be noted that multiplicative interaction does not necessarily imply additivity. Therefore, we also calculated the relative excess risk due to interaction (RERI) to quantify additive interactions. A RERI>0 indicates a positive additive interaction of air pollution and heat waves or low greenness on CVD risk, whereas an RERI<0 suggests a negative interaction, where the combined impact of exposures is less than the sum of their individual effects. We also calculated the attributable proportion (AP) and the synergy index (SI) to further characterise the interaction. The AP denotes the proportion of the disease risk in the doubly exposed group attributable to the interaction itself, rather than to the independent effects of the exposures. The SI is the ratio of the combined excess risk to the sum of the individual excess risks. An AP>0 or SI>1 indicates positive additive interaction. To address multiple testing for the primary hypotheses, we applied a Bonferroni-corrected threshold for the main effects of the seven environmental exposures (α = 0.05/7; approximately 0.007).

We conducted several sensitivity analyses to assess the robustness of our findings. First, we performed subgroup analyses by stratifying participants by age, sex, residence, education status, regional category, smoking status, cooking-fuel use, and CKM stage. Second, to account for potential lag effects in air quality changes, we assigned each participant to the prior year’s annual average pollutant concentration. Third, we excluded participants who experienced CVD events within two years of follow-up to minimise the potential for reverse causality. Lastly, we applied alternative definitions of heat waves, with varying threshold baselines, to examine the consistency of our results. We conducted all analyses using *R*, version 4.5.1 (R Core Team, Vienna, Austria).

## RESULTS

### Baseline characteristic

During follow-up, 1124 individuals developed incident CVD. Compared with participants without progression, participants who developed CVD were older with mean (x̄) age of 60.1 (standard deviation (SD) = 9.0) years *vs*. 56.6 (SD = 8.8) years (*P* < 0.001) and had higher BMI (*P* < 0.001), waist circumference (*P* < 0.001), triglycerides (*P* < 0.001), LDL-C (*P* < 0.001), fasting blood glucose (*P* < 0.001), and blood pressure (*P* < 0.001). They also had lower high-density lipoproteincholesterol levels, were less likely to have higher education, and were more often residents of midland regions. Besides, hypertension, diabetes, and hyperlipidaemia were more prevalent in the progression group (**Table 1**).

**Table 1:** Baseline characteristics of participants with CKM 0–3 stages*

Characteristics	With progression (n = 1124)	Without progression (n = 4503)	*P*-value
Age in years, x̄ (SD)	60.1 (9.0)	56.6 (8.8)	<0.001
BMI in kg/m^2^	24.0 (4.1)	23.3 (3.7)	<0.001
Waist circumference in cm, x̄ (SD)	85.9 (12.9)	83.3 (11.6)	<0.001
Sex			<0.001
*Female*	651 (57.9)	2314 (51.4)	
*Male*	473 (42.1)	2189 (48.6)	
Residence			0.886
*Rural*	752 (66.9)	3000 (66.6)	
*Urban*	372 (33.1)	1503 (33.4)	
Educational status			0.225
*Elementary school or below*	790 (70.3)	3078 (68.4)	
*Middle school or above*	334 (29.7)	1425 (31.6)	
Smoking status			0.463
*Non-smoker*	695 (61.8)	2728 (60.6)	
*Smoker*	429 (38.2)	1775 (39.4)	
Drinking status			<0.001
*Drinking but less than once a month*	78 (7.3)	372 (8.7)	
*Drinking more than once a month*	192 (18.1)	984 (23.1)	
*Non-drinker*	793 (74.6)	2911 (68.2)	
Regional category			0.006
*East*	354 (31.5)	1550 (34.4)	
*Midland*	450 (40.0)	1574 (35.0)	
*West*	320 (28.5)	1379 (30.6)	
Cooking fuel use			0.001
*Clean fuel*	333 (68.7)	1493 (76.2)	
*Solid fuel*	152 (31.3)	467 (23.8)	
Hypertension			<0.001
*No*	513 (45.6)	2731 (60.6)	
*Yes*	611 (54.4)	1772 (39.4)	
Diabetes			0.011
*No*	989 (88.0)	4079 (90.6)	
*Yes*	135 (12.0)	424 (9.4)	
Lipid			0.004
*No*	850 (75.6)	3584 (79.6)	
*Yes*	274 (24.4)	919 (20.4)	
SES			<0.001
*High*	4 (0.5)	20 (0.6)	
*Low*	230 (26.9)	666 (20.1)	
*Low-middle*	420 (49.1)	1763 (53.3)	
*Upper-middle*	202 (23.6)	857 (25.9)	
Triglycerides	119.7 (56.1)	111.8 (55.7)	<0.001
LDL-C	121.1 (33.5)	117.7 (32.2)	0.001
HDL-C	51.3 (14.6)	52.7 (14.6)	0.007
Fasting blood glucose in mg/dL, x̄ (SD)	108.3 (28.0)	105.3 (23.3)	<0.001
SBP in mmHg, x̄ (SD)	133.6 (22.3)	126.8 (19.9)	<0.001
DBP in mmHg, x̄ (SD)	76.8 (12.4)	74.6 (11.8)	<0.001
NO_2_, x̄ (SD)	31.8 (10.9)	30.0 (10.0)	<0.001
O_3_, x̄ (SD)	84.6 (5.2)	85.2 (5.4)	0.004
PM_1_, x̄ (SD)	33.9 (10.2)	32.2 (9.3)	<0.001
PM_10_, x̄ (SD)	103.3 (31.6)	95.8 (30.1)	<0.001
PM_2.5_, x̄ (SD)	61.0 (18.9)	57.4 (17.8)	<0.001
NDVI, x̄ (SD)	0.27 (0.093)	0.28 (0.082)	<0.001
Heat wave (92.5th percentile), x̄ (SD)	22.5 (25.0)	16.8 (21.3)	<0.001

DBP – diastolic blood pressure, HDL-C – high-density lipoprotein cholesterol, LDL-C – low-density lipoprotein cholesterol, NDVI – normalised difference vegetation index, NO_2_ – nitrogen dioxide, O_3_ – ozone, PM – particulate matter, SBP – systolic blood pressure, SD – standard deviation, SES – socioeconomic status, x̄ – mean

*Presented as n (%) unless specified otherwise.

Ambient air pollutant levels, NO_2_, PM_1_, PM_2.5_, and PM_10_, differed significantly between groups. Participants who developed CVD during follow-up were exposed to higher concentrations of air pollutants compared with those who remained at CKM stages 0–3 (PM_1_: x̄ = 33.9 (SD = 10.2) *vs*. x̄ = 32.2 (SD = 9.3), *P* < 0.001; PM_2.5_: x̄ = 61.0 (SD = 18.9) *vs*. x̄ = 57.4 (SD = 17.8), *P* < 0.001; PM_10_: x̄ = 103.3 (SD = 31.6) *vs*. x̄ = 95.8 (SD = 30.1), *P* < 0.001; NO_2_: x̄ = 31.8 (SD = 10.9) *vs.* x̄ = 30.0 (SD = 10.0), *P* < 0.001). Regarding greenness and heat-wave exposure, participants who progressed to CVD exhibited lower NDVI values and more frequent heat waves. In 2011, the mean NDVI was 0.27 (SD = 0.09) in the progression group compared with 0.28 (SD = 0.08) in the non-progression group (*P* < 0.001). Moreover, participants who developed CVD experienced more heat wave events (x̄ = 22.5 (SD = 25.0) *vs.* x̄ = 16.8 (SD = 21.3); *P* < 0.001).

### Association of air pollution exposure and CVD risk

**Figure 1:**
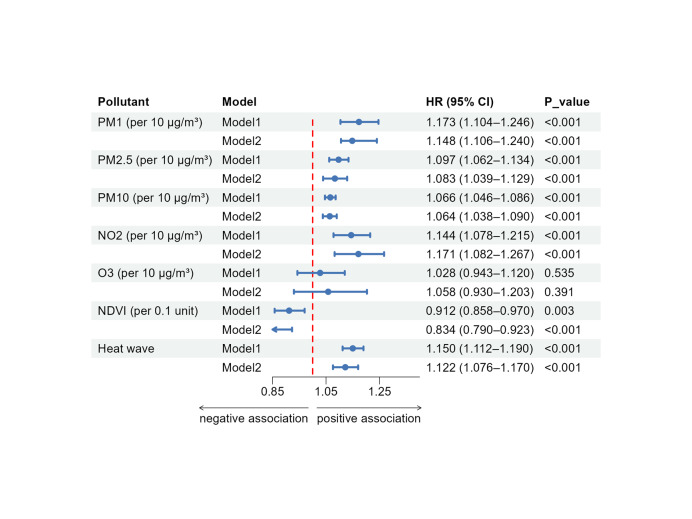
HRs of CVD risk in the CKM 0–3 participants associated with environmental exposures. Air pollutants were modelled per 10 μg/m^3^ increment, NDVI per 0.1-unit increase, and heat waves (≥92.5th percentile over three days) per additional event. The red dashed line represents the null value (HR = 1.0). Model 1 is unadjusted, while model 2 is fully adjusted for age, sex, education level, residence, regional category, smoking status, drinking status, and socioeconomic status.

A total of 1124 participants developed CVD during a follow-up time of seven years. Exposure to higher levels of particulate matter (PM_1_, PM_2.5_, and PM_10_) and NO_2_ was associated with an increased risk of future CVD incidence in participants with CKM stages 0–3 (**Figure 1**). For every 10μg/m^3^ increase in PM_1_, the CVD risk increased by 11.7% (HR = 1.173; 95% confidence interval (CI) = 1.104, 1.246, *P* < 0.001). Similarly, increases of 9.7%, 6.6%, and 14.4% in CVD risk were observed for each 10μg/m^3^ rise in PM_2.5_ (HR = 1.097; 95% CI = 1.062, 1.134, *P* < 0.001), PM_10_ (HR = 1.066; 95% CI = 1.046, 1.086, *P* < 0.001), and NO_2_ (HR = 1.144; 95% CI = 1.078, 1.215, *P* < 0.001), respectively. We did not note a significant association between O_3_ exposure and future CVD risk (HR = 1.028; 95% CI = 0.943, 1.120, *P* = 0.535). Even in the fully adjusted model, PM_1_ (HR = 1.148; 95% CI = 1.106, 1.240, *P* < 0.001), PM_2.5_ (HR = 1.083; 95% CI = 1.039, 1.129, *P* < 0.001), PM_10_ (HR = 1.064; 95% CI = 1.038, 1.090, *P* < 0.001), and NO_2_ (HR = 1.171; 95% CI = 1.082, 1.267, *P* < 0.001) remained positively associated with CVD risk in the CKM stage 0–3 population.

Subgroup analyses revealed significant heterogeneity in the association between PM exposure and CVD risk across different regional categories, educational status, CKM stages, and environmental context, suggesting potential effect modification (Figures S2–7 in the **Online Supplementary Document**). Participants with lower educational attainment, residing in the east or south part of China, and those at more advanced CKM stages demonstrated a higher risk of developing CVD (*P* < 0.05). PM-related CVD risk was higher in areas with lower NDVI, and was amplified under higher heat wave frequency (*P* < 0.05). By contrast, we observed no meaningful heterogeneity by age, sex, and residence (*P* > 0.05). These patterns were broadly consistent across PM_1_, PM_2.5_, and PM_10_.

### Association of heat waves, NDVI exposure, and CVD risk

Increased exposure to heat waves was associated with a higher incidence of CVD in the CKM population (HR = 1.15; 95% CI = 1.112, 1.190, *P* < 0.001). The effect of heat wave exposure on CVD risk in the CKM population was significantly modified by NDVI levels (*P* < 0.001). Participants residing in areas with low NDVI (HR = 1.29; 95% CI = 1.20, 1.37, *P* < 0.001) had a higher risk of CVD during heat wave events compared with those in areas with high NDVI (HR = 1.10; 95% CI = 1.09, 1.10, *P* < 0.001).

A 0.1 unit increase in NDVI was associated with a 9% lower risk of CVD (HR = 0.912; 95% CI = 0.858, 0.97, *P* = 0.003). Subgroup analysis did not identify any significant modifications to the relationship between NDVI exposure and CVD risk (Tables S4–5 in the **Online Supplementary Document**).

### Additive interaction between air pollution, heat waves, NDVI, and CVD risk

Our analysis indicated positive interactions between heat waves and air pollutants on the additive scale (**Table 2**). The relative excess risk due to RERI values of heat waves with air pollutants were significant for PM_1_ (RERI = 0.112; 95% CI = 0.033, 0.191), PM_2.5_ (RERI = 0.082; 95% CI = 0.020, 0.144), PM_10_ (RERI = 0.098; 95% CI = 0.019, 0.178), and NO_2_ (RERI = 0.081; 95% CI = 0.017, 0.145), while no significant interaction was observed with O_3_ (RERI = –0.005; 95% CI = –0.029, –0.018). Correspondingly, the AP was 6–9% for PM and NO_2_, and the SI ranged from 1.14 to 1.16, further supporting evidence of synergistic effects.

**Table 2:** Additive interaction effects of exposure to air pollution, heat waves, and NDVI on CKM 0–3 participants

Items	Heat wave			NDVI		
	**RERI (95% CI)**	**AP (95% CI)**	**SI (95% CI)**	**RERI (95% CI)**	**AP (95% CI)**	**SI (95% CI)**
PM_1_	0.112 (0.033, 0.191)	0.062 (0.028, 0.095)	1.158 (1.090, 1.225)	–0.075 (–0.144, –0.007)	–0.058 (–0.113, –0.003)	0.803 (0.610, 0.995)
PM_2.5_	0.082 (0.020, 0.144)	0.049 (0.200, 0.079)	1.140 (1.083, 1.198)	–0.051 (–0.105, –0.038)	–0.042 (–0.088, –0.005)	0.811 (0.585, 0.937)
PM_10_	0.098 (0.019, 0.178)	0.055 (0.020, 0.091)	1.144 (1.072, 1.217)	–0.097 (–0.176, –0.019)	–0.073 (–0.13, –0.011)	0.775 (0.587, 0.964)
NO_2_	0.081 (0.017, 0.145)	0.049 (0.019, 0.079)	1.142 (1.085, 1.199)	–0.036 (–0.083, 0.011)	–0.029 (–0.068, 0.011)	0.873 (0.682, 1.064)
O_3_	–0.005 (–0.029, 0.018)	–0.005 (–0.026, 0.016)	0.970 (0.820, 1.120)	–0.001 (–0.005, 0.004)	–0.001 (–0.005, 0.004)	1.051 (0.124, 1.978)

AP – attributable proportion due to interaction, CI – confidence interval, NDVI – normalised difference vegetation index, NO_2_ – nitrogen dioxide, O_3_ – ozone, PM – particulate matter, RERI – relative excess risk due to interaction, SI – synergy index

By contrast, we observed negative interactions between NDVI and particulate matter. Negative RERI values were observed for PM_1_ (RERI = –0.075; 95% CI = –0.144, –0.007), PM_2.5_ (RERI = –0.051; 95% CI = –0.105, –0.038), and PM_10_ (RERI = –0.097; 95% CI = –0.176, –0.019), suggesting that the excess risk associated with PM was lower in the presence of higher NDVI. The corresponding AP = –0.04, –0.07 and SI<1 reinforced the buffering effect of residential greenness. We did not find significant interactions between NDVI and NO_2_ or O_3_.

### Multiplicative interaction between air pollution, heat waves, NDVI, and CVD risk

**Figure 2:**
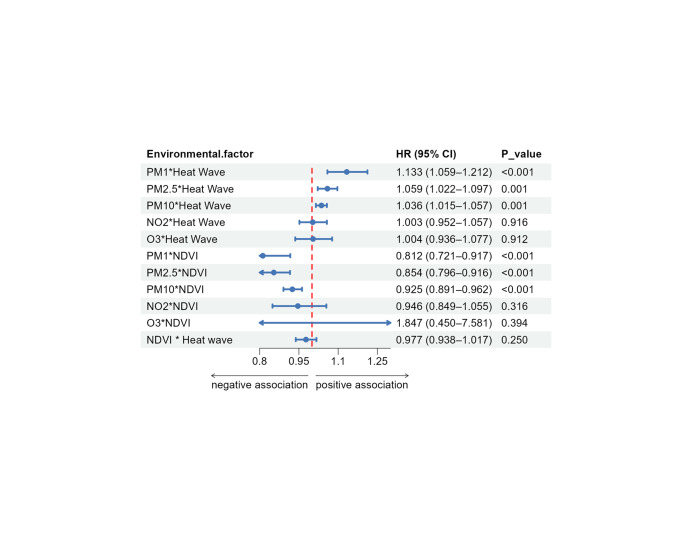
Multiplicative interactions between air pollution, heat waves, and NDVI in relation to CVD risk among participants with CKM stages 0–3. HRs and 95% CIs were estimated using time-varying Cox proportional hazards models, adjusted for age, sex, residence, education level, regional category, smoking status, drinking status, cooking fuel use, and socioeconomic status.

We further evaluated the multiplicative interactions between air pollution, heat waves, and NDVI on CVD risk in the CKM population (**Figure 2**). We observed significant positive interactions between heat waves and particulate matter. Specifically, the combined effect of heat waves and PM_1_ (HR = 1.133; 95% CI = 1.059, 1.212, *P* < 0.001), PM_2.5_ (HR = 1.059; 95% CI = 1.022, 1.097, *P* = 0.001), and PM_10_ (HR = 1.036; 95% CI = 1.015, 1.057, *P* = 0.001) was associated with increased CVD risk (Table S6 in the **Online Supplementary Document**). By contrast, we did not observe a significant multiplicative interaction for NO_2_ or O_3_ with heat waves (*P* > 0.05).

The NDVI showed a protective effect against particulate matter. Higher greenness attenuated the harmful effects of PM_1_ (HR = 0.812; 95% CI = 0.721, 0.917, *P* < 0.001), PM_2.5_ (HR = 0.854; 95% CI = 0.796, 0.916, *P* < 0.001), and PM_10_ (HR = 0.925; 95% CI = 0.891, 0.962, *P* < 0.001). No significant interactions were observed between NDVI and NO_2_ or O_3_. Additionally, NDVI did not significantly modify the effect of heat waves on CVD risk (HR = 0.977; 95% CI = 0.938, 1.017, *P* = 0.250).

**Figure 3:**
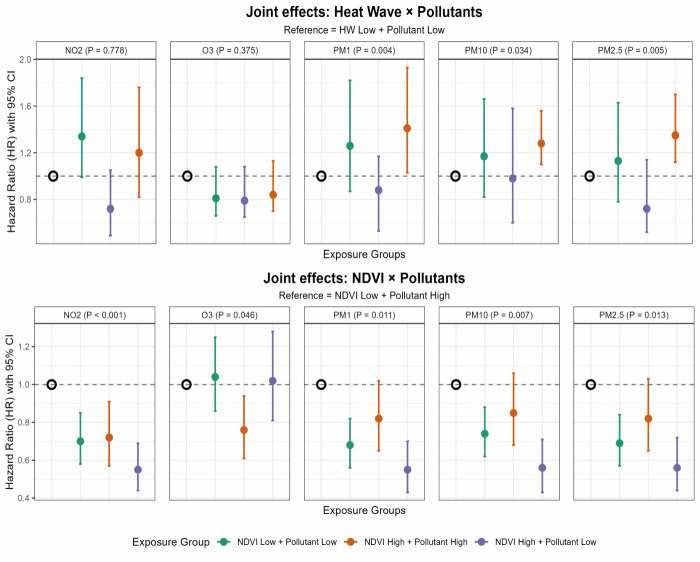
Interaction effects of air pollution, heat waves and NDVI on CVD risk in CKM 0–3 participants.

We analysed these variables as categorical measures to explore their relationships. CKM individuals concurrently exposed to high PM levels and heat waves exhibited a significantly increased risk of CVD (**Figure 3**). We observed a substantial reduction in CVD risk among participants simultaneously exposed to high NDVI and low PM levels, compared with those exposed to low NDVI and high PM levels. Importantly, even in areas with low NDVI, lower air pollution exposure still conferred a protective effect on CVD risk. These findings were consistent with additive interaction results (**Table 2**), supporting that heat wave amplifies PM effects, whereas NDVI buffers them.

## DISCUSSION

By linking CHARLS to Chinese environmental data, we identified significant associations between environmental exposures and CVD incidence in the CKM population. Higher levels of PM_1_, PM_2.5_, PM_10_, and NO_2_, as well as more frequent heat waves, were associated with increased CVD risk, whereas greater residential greenness was associated with reduced risk. These associations persisted after full adjustment for confounders. Subgroup analyses demonstrated heterogeneity across education, region, and CKM stage. Notably, air pollution and heat waves synergistically increased CVD risk, whereas higher NDVI mitigated these effects.

The association between air pollution and CVD risk was first recognised by the American Heart Association in 2004 [16], and has since been consistently confirmed across populations. Evidence indicates that individuals with pre-existing cardiometabolic conditions are particularly susceptible [17,18], aligning with our findings. A previous CHARLS-based study also reported significant associations for particulate matter and NO_2_ (PM_1_: HR = 1.09; PM_2.5_: HR = 1.04; PM_10_: HR = 1.05; NO_2_: HR = 1.11), but not O_3_, while our observed HRs were higher, likely due to the focus of participants with CKM who are more vulnerable. In subgroup analyses, PM_1_, PM_2.5_, PM_10_, and NO_2_ significantly interacted with CKM stages, with individuals in stages two and three exhibiting particularly elevated risks. The relationship between O_3_ exposure and CVD risk remains intriguing, with previous studies yielding heterogeneous results [19–21]. Several factors may explain the divergent findings. First, ambient O_3_ exhibits significant spatial and temporal variability, which complicates accurate exposure assessment. Second, meteorological conditions, including sunlight intensity, humidity, and temperature, can strongly influence both O_3_ formation and cardiovascular physiology, potentially modifying the observed associations [1].

The association between heat wave exposure and an increased risk of cardiovascular events, particularly acute conditions such as acute coronary syndrome, heart failure, and stroke, has been extensively documented in the literature [22]. Consistent with our findings, heat wave exposure was independently associated with an increased risk of CVD among individuals with CKM, and these associations remained robust across different definitions of heat waves (Table S7 in the **Online Supplementary Document**). The NDVI, an established indicator of residential greenness, has been linked to a wide range of health benefits [2]. In our study, each 0.1-unit increase in NDVI was associated with an approximately 9% reduction in CVD risk among individuals with CKM.

Beyond their independent effects, we identified significant interactions among air pollution, heat waves, and greenness on both additive and multiplicative scales. Positive RERI values indicated synergistic effects between heat waves and particulate matter or NO_2_, showing that concurrent exposure amplified CVD risk beyond the sum of individual effects. Conversely, negative RERI values for NDVI–PM interactions suggest that high residential greenness may partially offset the population-level risk associated with air pollution. On the multiplicative scale, heat waves and particulate matter jointly increased CVD hazard, whereas NDVI attenuated these associations (Table S6 in the **Online Supplementary Document**). Collectively, these findings highlight that extreme heat exacerbates, and residential greenness mitigates, the cardiovascular risk of air pollution. Our focus on additive interaction (RERI, AP, and SI) is particularly relevant for public health decision-making. While multiplicative models are convenient for statistical estimation, the additive scale corresponds more directly to the absolute number of disease cases in a population [23]. A positive additive interaction (RERI>0 and SI>1) implies that the combined impact of two exposures exceeds the simple sum of their individual effects. In our study, the significant AP indicates that a substantial fraction of CVD cases is attributable to the co-occurrence of air pollution and heat waves. Similarly, the negative interaction with greenness implies that the absolute risk of air pollution is disproportionately higher in areas with low vegetation. These findings indicate statistical interaction on the additive scale, rather than mechanistic synergy or buffering, and should be interpreted accordingly.

Several biological pathways may plausibly underlie the observed statistical interactions; however, caution is warranted when inferring causality. Extreme heat may amplify the physiological stress associated with air pollution exposure through multiple, interrelated processes. Regarding the mitigating role of greenness, NDVI should be interpreted as a composite indicator that captures a range of potential protective effects, including air-pollution attenuation, heat-island mitigation, increased physical activity, and reduced psychosocial stress. Importantly, our analysis does not permit attribution of the observed protective associations to any single pathway, and future studies incorporating mediation analyses are needed to clarify the specific mechanisms.

Subgroup analyses suggested potential effect modification by educational attainment: stronger associations between particulate matter exposure and CVD risk were observed among individuals with lower educational attainment, whereas effects were attenuated and nonsignificant among those with higher educational attainment. These patterns may reflect underlying socioeconomic vulnerability, including differential access to healthcare and protective resources [24]. Geographic disparities were also evident: participants in eastern China showed greater susceptibility to air pollution than those in other regions, consistent with previous evidence linking industrialisation, population density, and energy use to intensified exposure and health impacts. Moreover, the cardiovascular risks related to heat waves were mitigated by residential greenness, as shown in stratified analyses. However, given the number of subgroup comparisons conducted, these findings should be interpreted cautiously and regarded as exploratory and hypothesis-generating rather than confirmatory.

Multiple sensitivity analyses confirmed the robustness of our findings. Stratified analyses by urban-rural residence and major geographic regions partially addressed potential exposure misclassification; consistent regional effects were observed despite non-significant stratification by residence type. Excluding participants with CVD before 2013 and using one-year-lagged exposures yielded similar results. Alternative heat-wave definitions (using varying temperature percentiles and minimum durations) yielded comparable estimates. These findings reinforce the stability and validity of the main associations despite acknowledged spatial-resolution limitations. The implementation of the China Clean Air Action policy (2013–2017) substantially reduced PM_2.5_ and PM_10_ emissions, and prior quasi-experimental and meta-analytic studies have shown that such interventions yield significant cardiovascular health benefits [25–27]. In an exploratory descriptive analysis, we compared crude CVD incidence rates between cities that met *vs.* did not meet the China Clean Air Action targets. We observed that participants in cities that met the targets generally had lower CVD incidence rates than those in non-compliant cities (Figure S4 in the **Online Supplementary Document**). However, we emphasise that these comparisons are purely descriptive. Due to data limitations in the CHARLS survey, a rigorous difference-in-differences analysis was not feasible to control for pre-existing trends.

Compared with previous studies that used average exposures over follow-up [28,29], we incorporated time-varying covariates into Cox models, thereby reducing temporal exposure misclassification in long-term cohorts, particularly given the substantial environmental changes in China following the Clean Air Action Plan in 2013 (Figure S5 in the **Online Supplementary Document**) [30]. Nonetheless, limitations exist. First, due to privacy restrictions, exposures were assigned at the city level. While this spatial resolution is generally appropriate for regionally homogeneous air pollutants, it may lead to exposure misclassification for more spatially heterogeneous exposures, such as NDVI and heat waves [31], for which within-city variability is substantial. In addition, exposure estimates were derived from different data sources: PM_1_ and PM_2.5_ from modelled data and PM_10_, NO_2_, and O_3_ from fixed-site monitoring, which may introduce heterogeneous measurement error across pollutants. Such spatial and source-related measurement errors are likely non-differential and may bias effect estimates toward the null; therefore, the observed positive associations likely represent conservative estimates of the true risks [32]. Nevertheless, comparisons of cross-pollutant effect magnitudes should be interpreted with caution. Future studies using high-resolution residential data are warranted. Second, CVD events were self-reported; to mitigate potential recall bias, we adjusted for regional category, socioeconomic status, and education. Third, because CHARLS primarily includes middle-aged and older adults and reflects pollution mixtures specific to China, our findings may not be generalizable to younger populations or to urban settings in Western countries. Furthermore, high multicollinearity among pollutants (pairwise correlation coefficients >0.85) (Figure S6 in the **Online Supplementary Document**) precluded the use of multi-pollutant models. Consequently, our single-pollutant estimates should be interpreted with caution as they may reflect the combined effects of correlated components within the broader ambient pollution mixture.

## CONCLUSIONS

Our study demonstrates that long-term exposure to air pollution and heat waves is independently associated with an increased risk of incident CVD among adults with CKM syndrome, whereas residential greenness is associated with a reduced risk. Beyond these main effects, we observed significant interactions on the additive scale. Specifically, the joint association of heat waves and particulate matter with CVD risk exceeded the sum of their individual risks, while higher greenness levels showed negative interactions with air pollution. From a public health perspective, these deviations from additivity imply that air pollution control strategies may yield the greatest absolute reduction in CVD cases if prioritised during periods of extreme heat or targeted at communities with limited green space. Stratified analyses further indicated that individuals with lower educational attainment and those at advanced baseline CKM stages may be particularly susceptible.

## Additional material


Online Supplementary Document

## References

[1] Ndumele CE Neeland IJ Tuttle KR Chow SL Mathew RO Khan SS A Synopsis of the Evidence for the Science and Clinical Management of Cardiovascular-Kidney-Metabolic (CKM) Syndrome: A Scientific Statement From the American Heart Association. Circulation. 2023;148:1636–64. 10.1161/CIR.0000000000001186 37807920

[2] Ndumele CE Rangaswami J Chow SL Neeland IJ Tuttle KR Khan SS Cardiovascular-Kidney-Metabolic Health: A Presidential Advisory From the American Heart Association. Circulation. 2023;148:1606–35. 10.1161/CIR.0000000000001184 37807924

[3] Rajagopalan S Landrigan PJ Pollution and the Heart. N Engl J Med. 2021;385:1881–92. 10.1056/NEJMra2030281 34758254

[4] Peng S Li Z Ji JS Chen B Yin X Zhang W Interaction between Extreme Temperature Events and Fine Particulate Matter on Cardiometabolic Multimorbidity: Evidence from Four National Cohort Studies. Environ Sci Technol. 2024;58:12379–89. 10.1021/acs.est.4c02080 38961056 PMC11256764

[5] Xu R Huang S Shi C Wang R Liu T Li Y Extreme Temperature Events, Fine Particulate Matter, and Myocardial Infarction Mortality. Circulation. 2023;148:312–23. 10.1161/CIRCULATIONAHA.122.063504 37486993

[6] Lu C Zhang Y Li B Zhao Z Huang C Zhang X Interaction effect of prenatal and postnatal exposure to ambient air pollution and temperature on childhood asthma. Environ Int. 2022;167:107456. 10.1016/j.envint.2022.107456 35952466

[7] Sharma R Schinasi LH Lee BK Weuve J Weisskopf MG Sheffield PE Air Pollution and Temperature in Seizures and Epilepsy: A Scoping Review of Epidemiological Studies. Curr Environ Health Rep. 2024;12:1. 10.1007/s40572-024-00466-3 39656387 PMC11631820

[8] Rancière F Wafo O Perrot X Momas I Associations between heat wave during pregnancy and term birth weight outcomes: The PARIS birth cohort. Environ Int. 2024;188:108730. 10.1016/j.envint.2024.108730 38776654

[9] Remigio RV He H Raimann JG Kotanko P Maddux FW Sapkota AR Combined effects of air pollution and extreme heat events among ESKD patients within the Northeastern United States. Sci Total Environ. 2022;812:152481. 10.1016/j.scitotenv.2021.152481 34921874 PMC8962569

[10] Fan S Hu H Li Y Deng Y Wang S Xian J Effects of ambient air pollution mixtures and household fuel use on progression to advanced cardiovascular-kidney-metabolic syndrome among middle-aged and older adults. Ecotoxicol Environ Saf. 2025;303:118915. 10.1016/j.ecoenv.2025.118915 40850115

[11] Rudan I Song P Adeloye D Campbell H Journal of Global Health’s Guidelines for Reporting Analyses of Big Data Repositories Open to the Public (GRABDROP): preventing ‘paper mills’, duplicate publications, misuse of statistical inference, and inappropriate use of artificial intelligence. J Glob Health. 2025;15:01004. 10.7189/jogh.15.01004 40587200 PMC12208284

[12] Chen G Knibbs LD Zhang W Li S Cao W Guo J Estimating spatiotemporal distribution of PM(1) concentrations in China with satellite remote sensing, meteorology, and land use information. Environ Pollut. 2018;233:1086–94. 10.1016/j.envpol.2017.10.011 29033176

[13] Chen G Li S Knibbs LD Hamm NAS Cao W Li T A machine learning method to estimate PM(2.5) concentrations across China with remote sensing, meteorological and land use information. Sci Total Environ. 2018;636:52–60. 10.1016/j.scitotenv.2018.04.251 29702402

[14] Chen G Wang Y Li S Cao W Ren H Knibbs LD Spatiotemporal patterns of PM(10) concentrations over China during 2005-2016: A satellite-based estimation using the random forests approach. Environ Pollut. 2018;242:605–13. 10.1016/j.envpol.2018.07.012 30014938

[15] Chen G Li Y Zhou Y Shi C Guo Y Liu Y The comparison of AOD-based and non-AOD prediction models for daily PM(2.5) estimation in Guangdong province, China with poor AOD coverage. Environ Res. 2021;195:110735. 10.1016/j.envres.2021.110735 33460631

[16] Brook RD Rajagopalan S Pope CA III Brook JR Bhatnagar A Diez-Roux AV Particulate matter air pollution and cardiovascular disease: An update to the scientific statement from the American Heart Association. Circulation. 2010;121:2331–78. 10.1161/CIR.0b013e3181dbece1 20458016

[17] Yang BY Guo Y Markevych I Qian ZM Bloom MS Heinrich J Association of Long-term Exposure to Ambient Air Pollutants With Risk Factors for Cardiovascular Disease in China. JAMA Netw Open. 2019;2:e190318. 10.1001/jamanetworkopen.2019.0318 30848806 PMC6484675

[18] Xu X Tang L Kang Y Liang J Geng W Wang Y Association of air pollutions and systemic inflammation with early cardiovascular-kidney-metabolic syndrome among middle-aged and elderly adults: a cross-sectional study from CHARLS. Sci Rep. 2025;15:39377. 10.1038/s41598-025-24690-5 41214161 PMC12603026

[19] Jerrett M Burnett RT Pope CA III Ito K Thurston G Krewski D Long-term ozone exposure and mortality. N Engl J Med. 2009;360:1085–95. 10.1056/NEJMoa0803894 19279340 PMC4105969

[20] Carey IM Atkinson RW Kent AJ van Staa T Cook DG Anderson HR Mortality associations with long-term exposure to outdoor air pollution in a national English cohort. Am J Respir Crit Care Med. 2013;187:1226–33. 10.1164/rccm.201210-1758OC 23590261 PMC3734610

[21] Liang S Chen Y Sun X Dong X He G Pu Y Long-term exposure to ambient ozone and cardiovascular diseases: Evidence from two national cohort studies in China. J Adv Res. 2024;62:165–73. 10.1016/j.jare.2023.08.010 37625570 PMC11331174

[22] Cheng J Xu Z Bambrick H Prescott V Wang N Zhang Y Cardiorespiratory effects of heatwaves: A systematic review and meta-analysis of global epidemiological evidence. Environ Res. 2019;177:108610. 10.1016/j.envres.2019.108610 31376629

[23] VanderWeele TJ On the distinction between interaction and effect modification. Epidemiology. 2009;20:863–71. 10.1097/EDE.0b013e3181ba333c 19806059

[24] Luo H Zhang Q Yu K Meng X Kan H Chen R Long-term exposure to ambient air pollution is a risk factor for trajectory of cardiometabolic multimorbidity: A prospective study in the UK Biobank. EBioMedicine. 2022;84:104282. 10.1016/j.ebiom.2022.104282 36174399 PMC9520206

[25] Zou B You J Lin Y Duan X Zhao X Fang X Air pollution intervention and life-saving effect in China. Environ Int. 2019;125:529–41. 10.1016/j.envint.2018.10.045 30612707

[26] Yorifuji T Kashima S Doi H Fine-particulate Air Pollution from Diesel Emission Control and Mortality Rates in Tokyo: A Quasi-experimental Study. Epidemiology. 2016;27:769–78. 10.1097/EDE.0000000000000546 27479647

[27] Shah AS Langrish JP Nair H McAllister DA Hunter AL Donaldson K Global association of air pollution and heart failure: a systematic review and meta-analysis. Lancet. 2013;382:1039–48. 10.1016/S0140-6736(13)60898-3 23849322 PMC3809511

[28] Zhao L Zhao C Sun W Zheng H Gao Y Wa CK Long-term air pollution exposure and cardiovascular disease risk across cardiovascular-renal-metabolic stages: a nationwide study. BMC Public Health. 2025;25:2179. 10.1186/s12889-025-23348-1 40604548 PMC12220752

[29] Gui C Xiao Z Li M Xu J Air pollution promotes the onset and progression of cardiovascular-kidney-metabolic syndrome: a nationwide prospective cohort study. BMC Public Health. 2025;25:4287. 10.1186/s12889-025-25578-9 41437340 PMC12729196

[30] Mansournia MA Etminan M Danaei G Kaufman JS Collins G Handling time varying confounding in observational research. BMJ. 2017;359:j4587. 10.1136/bmj.j4587 29038130

[31] Liu Z Cai M Wu D Yu P Jiao Y Jiang Q Effects of nanoplastics at predicted environmental concentration on Daphnia pulex after exposure through multiple generations. Environ Pollut. 2020;256:113506. 10.1016/j.envpol.2019.113506 31706756

[32] Thacher JD Poulsen AH Hvidtfeldt UA Raaschou-Nielsen O Brandt J Geels C Long-Term Exposure to Transportation Noise and Risk for Type 2 Diabetes in a Nationwide Cohort Study from Denmark. Environ Health Perspect. 2021;129:127003. 10.1289/EHP9146 34855467 PMC8638828

