# Supplementary material for: Environmental determinants of cardiovascular-kidney-metabolic health: interactive roles of air pollution, heat waves, and green spaces
Source: J Glob Health. 2026 Feb 27;16:04073. doi: 10.7189/jogh.16.04073 (PMC12945347; doi:10.7189/jogh.16.04073)
Supplement: Online Supplementary Document [file jogh-16-04073-s001.zip › jogh-16-04073-s001.pdf]

Supplement to: Li A, Fang C, Li Y. Environmental determinants of cardiovascular-kidney-metabolic health: interactive roles of air pollution, heat waves, and green spaces. J Glob Health. 2026;16:04073.

Figure S1. Participants enrollment flowchart

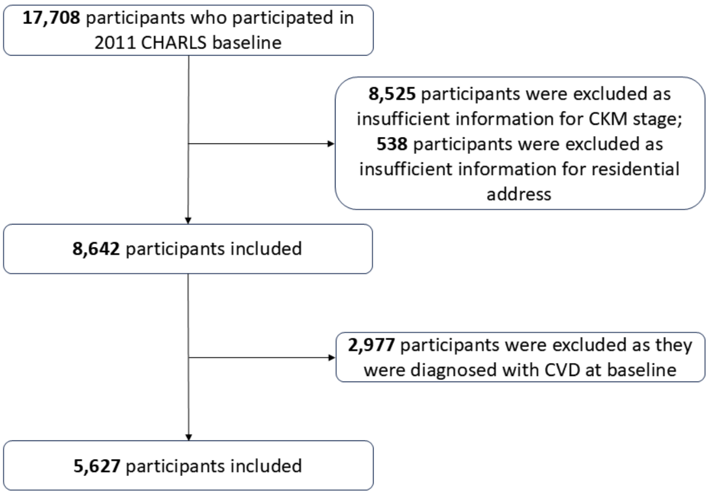

Figure S2: Subgroup analysis of the relationship between air pollutants and CVD risk in the CKM 0-3 participants

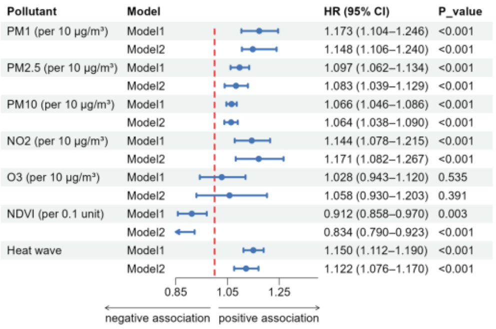

Separate analyses were conducted for each pollutant, with each model adjusted for age, sex, residence, education level, regional category, drinking status, smoking status, cooking fuel use and SES. The P for interaction indicates whether the stratification variable significantly modified the association between each pollutant and CVD risk.

Figure S3: Subgroup analysis of the relationship between heat waves, NDVI, and CVD risk in the CKM 0-3 participants

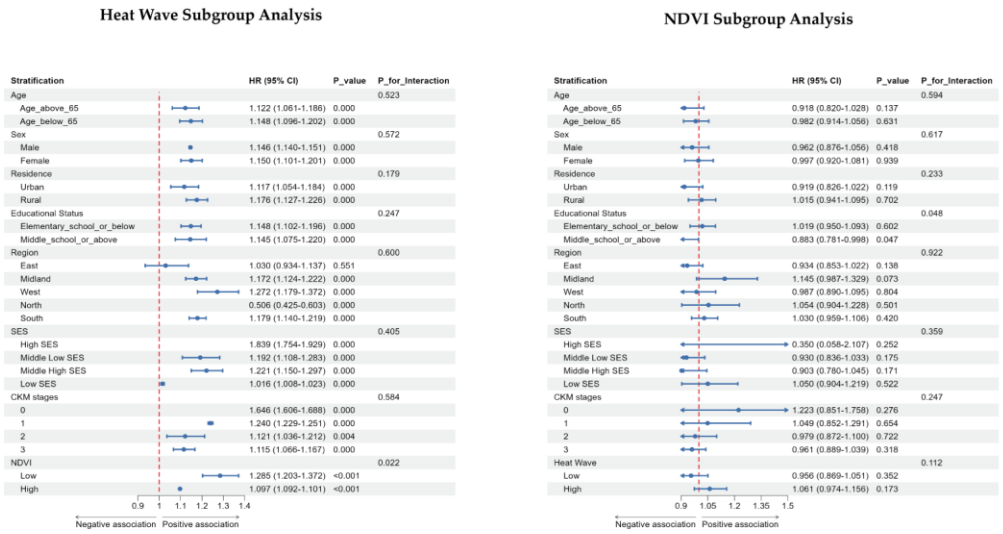

Each model was adjusted for age, sex, residence, education level, regional category, drinking status, smoking status, cooking fuel use, and CKM stages. Separate analyses were conducted for each pollutant, with each model adjusted for age, sex, residence, education level, regional category, drinking status, smoking status, cooking fuel uses and SES. The P for interaction indicates whether the stratification variable significantly modified the association between each pollutant and CVD risk.

Figure S4: Kaplan-Meier curve comparison between CCAA-treated and control group

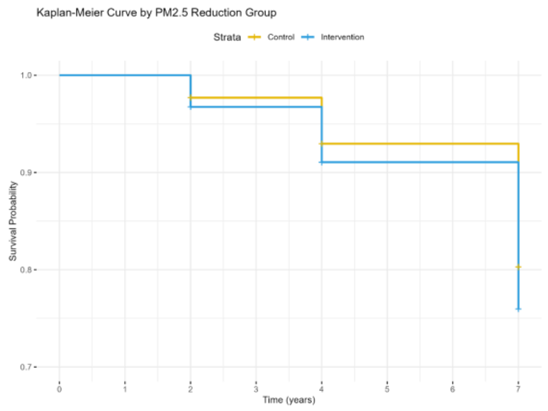

The analysis compared cumulative CVD incidence between participants residing in cities that achieved the CCAA reduction goals (improvement group) and those in cities that did not (non-improvement group). Although precise difference-in-difference analyses were not feasible due to the lack of province-level CVD incidence data, this approach provides an approximate evaluation of the potential health benefits of air quality improvements.

Figure S5: Air pollution comparison between 2011 and 2018

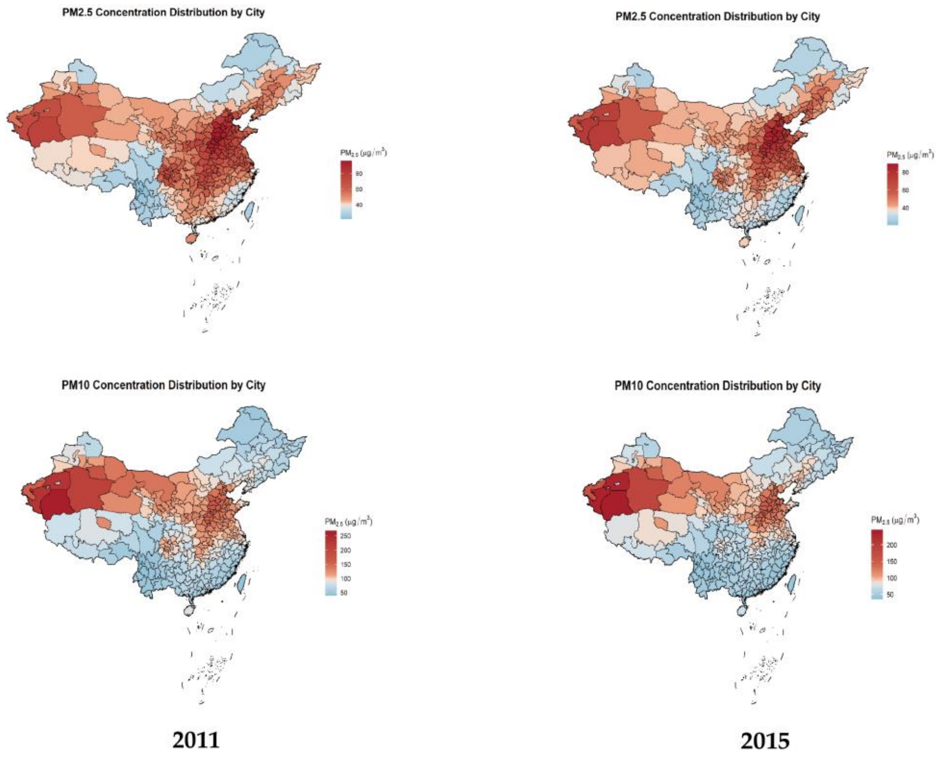

Maps illustrate the city-level average concentrations of $PM_{2.5}$ (top row) and PM_{10} (bottom row), showing marked spatial variation and temporal changes during the study period.

Figure S6: Correlation between environmental factors

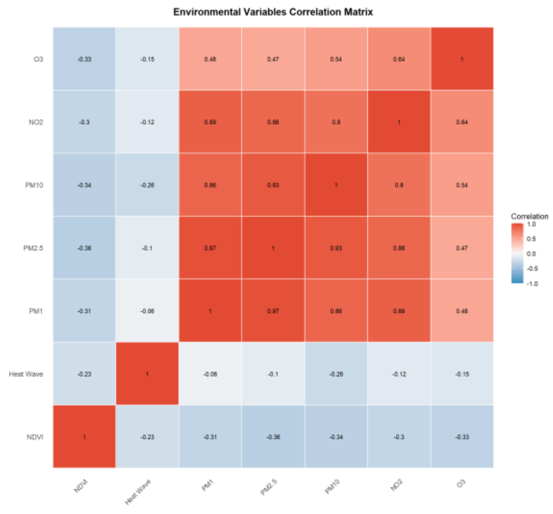

Time-Varying Cox Proportional Hazards Model

We employed a time-dependent Cox proportional hazards model to account for the dynamic nature of environmental covariates over time. This approach allows us to evaluate the impact of changing environmental exposures, such as air pollution and heat waves, on cardiovascular

risk while adjusting for baseline covariates that remain fixed over time.

The time-varying Cox model can be expressed as follows [1]:

$$h(t|Z_i(t), X_i) = h_0(t) * exp(\beta X_i + \gamma Z_i(t))$$

In this equation, Zi(t) denotes the time-varying exposure for individual i at time t, while Xi represents the time-fixed covariates at baseline, which include demographic factors (age, sex, urban/rural residence, co-residence, and marital status) and socioeconomic status (education and occupation). h(t|Zi(t),Xi) is the hazard function at time t for individual i, given the covariates Zi(t) and Xi· h0(t) is the baseline hazard function, stratified by province (denoted as prov), representing the residential province of the individual. The stratified Cox model allows for separate baseline hazard functions for different residential provinces. The coefficients β and γ represent the effects of the time-fixed and time-varying covariates, respectively.

Equations of REOI, AP, and SI

These three indicators were calculated using the following formulas [2]:

$$\text{REOI} = \frac{\text{OR}_{11} - 1}{\text{OR}_{10} - 1} + \frac{\text{OR}_{01} - 1}{\#1}; \text{AP} = \frac{\text{REOI}}{\text{OR}_{11}}; \quad \text{SI} = \frac{\text{OR}_{11} - 1}{\text{OR}_{10} - 1} + \frac{\text{OR}_{01} - 1}{\#3}$$

Where:

REOI denotes the effect due to interaction; RERI = 0 indicates that there is no interaction between two variables, and the combined exposure effect is directly equal to the sum of their exposure effects. RERI > 0 indicates that there is synergistic effect. RERI < 0 indicates that there is antagonistic effect.

AP represents the proportion of the combined effect attributable to interaction; AP = 0 indicates no interaction. AP > 0 indicates there is synergistic effect. AP < 0 indicates that there is antagonistic effect. The value of AP ranges from -1 to 1.

SI refers to the ratio of the combined effect to the individual effect. S = 1 indicates there is no interaction or; S >1 indicates there is synergistic effect. S < 1 indicates there is antagonistic effect. The values of S range from 0 to positive infinity.

OR10, OR01, OR11 represent the odds ratios (OR) for level 2, level 3, and level 4 compared with level 1 (OR_{00} =1), respectively.

If REOI > 0 (or AP > 0 or SI > 1), it indicates a synergistic interaction, meaning the combined effect is greater than the individual effects of $PM_{2.5}$ constituents and extreme temperature events (ETEs) on stroke mortality. Conversely, REOI < 0 (or AP < 0 or SI < 1) signifies an antagonistic interaction, where the combined effect is less than expected based on the individual effects.

References

1. Fisher, L.D. and D.Y. Lin, *Time-dependent covariates in the Cox proportional-hazards regression model.* Annu Rev Public Health, 1999. **20**: p. 145-57.
2. Knol, M.J., et al., *Estimating measures of interaction on an additive scale for preventive exposures.* Eur J Epidemiol, 2011. **26**(6): p. 433-8.
